# Optimisation of enzymatic hydrolysis of apple pomace for production of biofuel and biorefinery chemicals using commercial enzymes

**DOI:** 10.1007/s13205-015-0312-7

**Published:** 2015-06-20

**Authors:** Repson Gama, J. Susan Van Dyk, Brett I. Pletschke

**Affiliations:** Department of Biochemistry and Microbiology, Rhodes University, PO Box 94, Grahamstown, 6140 South Africa; Forest Products Biotechnology Group, University of British Columbia, 2424 Main Mall, Vancouver, BC V6T 1Z4 Canada

**Keywords:** Apple pomace, Celluclast, Inhibition, Lignocellulose, Viscozyme

## Abstract

Apple pomace, a waste product from the apple juice industry is a potential feedstock for biofuel and biorefinery chemical production. Optimisation of hydrolysis conditions for apple pomace hydrolysis using Viscozyme L and Celluclast 1.5L was investigated using 1 L reaction volumes. The effects of temperature, pH, β-glucosidase supplementation and substrate feeding regimes were determined. Hydrolysis at room temperature using an unbuffered system gave optimal performance. Reactors in batch mode resulted in a better performance (4.2 g/L glucose and 16.8 g/L reducing sugar, 75 % yield for both) than fed-batch (3.2 g/L glucose and 14.6 g/L reducing sugar, 65.5 and 73.1 % yield respectively) in 72 h. The addition of Novozyme 188 to the core mixture of Viscozyme L and Celluclast 1.5L resulted in the doubling of glucose released. The main products (yield %) released from apple pomace hydrolysis were galacturonic acid (78 %), glucose (75 %), arabinose (90 %) and galactose (87 %). These products are potential raw materials for biofuel and biorefinery chemical production.

## Introduction

Apple pomace is the waste produced from the extraction of juice from apples. Large quantities of waste are produced worldwide every year, with 25–35 % of the 70 million metric tonnes produced ending up as waste (Food and Agricultural Organisation of the United Nations 2012; Gullon et al. 2008), an estimated 17–24 million metric tonnes per year (Van Dyk et al. 2013). In most cases, the pomace is disposed of, which poses major environmental and health problems due to its high moisture content (70–75 %), high chemical oxygen demand (COD, 10,000 mg/L) and biological oxygen demand (BOD) (Bhushan et al. 2008; Capek et al. 1995; Mahmood et al. 2010; Parmar and Rupasinghe 2013). Combining treatment of this waste with production of value-added products can solve disposal problems, while potentially creating additional revenue in the bio-economy.

Apple pomace is lignocellulosic in nature and a rich source of cellulose, pectin and hemicellulose, which can be enzymatically converted to sugar monomers and other value-added products such as acetic acid, galacturonic acid, phenolics, vanillin, etc. (Bhushan et al. 2008; Foyle et al. 2007; Joshi and Attri 2006; Parmar and Rupasinghe 2013; Vendruscolo and Ninow 2014). Enzymatic degradation of lignocellulosic substrates requires a number of enzymes, working in synergy. Cellulose degradation requires cellobiohydrolases, endoglucanases and β-glucosidases. While hemicellulose and the enzymes required for its degradation have been studied, hemicellulose composition can be variable. In the case of apple pomace, the hemicellulose composition has not been studied and therefore it is not clear which enzymes are required. Some authors have indicated that apple pomace contains xylan (Bhushan et al. 2008; Dhillon et al. 2012; Parmar and Rupasinghe 2012), which would indicate a requirement for endoxylanases and β-xylosidases, as well as possible debranching enzymes. However, the xylose found in apple pomace could also be present in the form of xyloglucan or xylogalacturonan. Le Bourvellec et al. (2011) indicated that fucogalactoxyloglucan was the main hemicellulose in apple pomace. This could indicate a requirement for a completely different set of enzymes compared to xylan hydrolysis, for example xyloglucanases.

Apple pomace also contains high levels of pectin. However, studies on pectin degradation in complex substrates have been very limited. Pectin is highly complex and many enzymes have activity on pectin, for example methyl esterase, acetyl esterase, pectate lyase, pectin lyase, endo-polygalacturonase, exo-polygalacturonase, arabinase and galactanase (Van den Brink and de Vries 2011; Voragen et al. 2009). Pectinases usually refer to the enzymes that cleave the backbone to release galacturonic acid, while arabinase and galactanase cleave the neutral side chains in rhamnogalacturonan I into neutral sugars, arabinose and galactose.

Commercially available enzyme cocktails contain multiple enzymes which act on lignocellulose, with different cocktails optimised for different substrates. As there are no ready-made cocktails for apple pomace degradation, we investigated combinations of cocktails which together would contain the enzymes required for its hydrolysis.

## Materials and methods

### Apple pomace biomass preparation

Golden Delicious apples, obtained from a local supermarket, were cut into small pieces and the juice extracted using a domestic juicer to separate the pomace from the juice. The pomace was then homogenised in a food processor and washed several times with distilled water until no sugars were detected in the wash using the 3,5-dinitrosalicylic acid (DNS) assay (Miller 1959). The apple pomace was stored wet at −20 °C. Apple pomace was used at various substrate loadings based on the wet weight, with dry weight equivalent approximately 10 % of the wet weight. The dry weight was determined by freeze-drying the apple pomace. Sodium azide (0.03 % w/v) was used as a microbial preservative in buffers and apple pomace.

### Enzymes

Three commercial enzyme preparations were used in this study: Viscozyme L (“Viscozyme”) (an enzyme complex from *Aspergillus aculeatus*) (Sigma); Celluclast 1.5L (“Celluclast”) (a commercial *Trichoderma reesei* ATCC cellulase preparation) (Sigma); Biocip Membrane (“Biocip”) (Novozymes, Bagsvaerd, Denmark) and Novozyme 188 (a commercial *Aspergillus niger*—β-glucosidase preparation). Various stock concentrations were prepared in appropriate buffers depending on the application.

### Polysaccharide and lignin composition of apple pomace

The composition of apple pomace was characterised using a modified method by Moxley and Zhang (2007) and Sluiter et al. (2011).

#### Determination of sugar composition of apple pomace

Freeze-dried and finely ground apple pomace was hydrolysed using 72 % sulphuric acid according to the method by Moxley and Zhang (2007). The 4 and 1 % acid hydrolysate were centrifuged at 13 000*×* *g* for 5 min and the supernatant stored in the freezer. The 4 % hydrolysate was used to measure glucose, galactose, fructose and galacturonic acid and the 1 % hydrolysate to measure xylose and arabinose (using specific Megazyme kits and high performance liquid chromatography (HPLC) methods). HPLC was carried out using a Shimadzu HPLC (Shimadzu Scientific, Japan) equipped with a Refractive Index detector and Shodex column (8.0 mm ID × 300 mm L, SP-0810, Japan). The mobile phase was deionised water, with a flow rate of 1.0 mL/min and a column temperature of 80 °C.

Correction coefficients (CR) for each sugar were determined using known samples of each sugar that were treated in the same way as the samples above.

CR = Concentration measured/concentration known.

The concentration (*C*_s_, %) of the sugars in the polysaccharide was calculated as follows:

$$C_{\text{s}} = \, \left( {\left( {C_{\text{i}} V/{\text{CR}}} \right)*\left( {{\text{MW}}_{\text{p}} /{\text{MW}}_{\text{m}} } \right)/{\text{Wt}}} \right)* 100$$

where *C*_i_ represents the measured sugar concentration, MW_p_ the molecular weight of hexose or pentose polysaccharides (162.14 or 132.11 g/mol), MW_m_ the molecular weight of monomeric hexose or pentose (180.16 or 150.13 g/mol), Wt the weight of sample and *V* the volume of hydrolysate (mL).

#### Determination of acid insoluble lignin

Aliquots of 86 mL of the 4 % sulphuric acid hydrolysed samples were quantitatively transferred into filtering sintered crucibles, porosity 3, using 50 mL of warm deionised water. Filtration was performed and the solid residues were dried at 105 °C for 4 h or until a constant weight was achieved. Samples were then cooled in a desiccator. The weight was recorded (W1). The crucibles were then placed in a muffle furnace at 580 °C for 24 h, cooled in a desiccator and then weighed (W2). The amount of acid insoluble lignin was then calculated (W1–W2).

### Enzyme assays

The activity of the enzymes on apple pomace was determined by measuring reducing sugars released (as glucose equivalents) using a modified 3,5-dinitrosalicylic acid (DNS) assay method (Miller 1959). The DNS assay was performed as was described by Beukes et al. (2008). The concentration of reducing sugars released from apple pomace was determined as glucose equivalents using a glucose standard curve. Detection of individual sugars (glucose, xylose, arabinose and galacturonic acid) was performed using commercial kits (Megazyme, Ireland) according to the manufacturer’s instruction manual. Standard curves for each sugar were used to estimate the amount of sugar released.

The glucose and reducing sugar yields (%) from cellulose and total pomace, respectively, were calculated using the following formulas;

Glucose yield (%) = Glucose liberated (g) × 0.9 × 100/Initial cellulose (g)

Reducing sugar yield (%) = reducing sugar liberated (g) × 100/Initial polysaccharides (g)

Calculations were based on dry weight.

### Identification of enzyme activities in Viscozyme and Celluclast

The different enzyme activities in Viscozyme and Celluclast were determined using various substrates. Carboxymethyl cellulose was used for endoglucanase activity, birchwood xylan was used for endoxylanase, locust bean gum was used for endomannanase, polygalacturonic acid was used for polygalacturonase and apple pectin was used for pectinase activity. A 2 % (w/v) (0.2 dry weight/v) substrate stock solution in citrate buffer (pH 5.0, 0.05 M) was prepared. An enzyme concentration of 0.304 mg/mL stock solution was prepared for each enzyme in citrate buffer. The assay was performed in triplicate, under standard assay conditions at 37 °C for 30 min. Sugars were quantified using the DNS assay. Activity was estimated using a glucose standard for endoglucanase, a xylose standard curve for endoxylanase and endomannanase, and a galacturonic acid standard curve for polygalacturonase and pectinase. A modified 4-nitrophenol assay (Berghem and Pettersson 1974) was used to determine the activities of the other enzymes.

To determine the optimal ratio for Viscozyme (V) and Celluclast (C), various percentage ratios (in terms of protein) of the enzyme mixtures were assayed, while keeping the total protein concentration in the assay constant at 0.076 mg/mL. The following combinations were assayed: V100: C0; V87.5: C12.5; V75: C25; V62.5: C37.5; V50: C50; V37.5: C62.5; V25: C75; V12.5: C87.5; V0: C100. The assay was performed for 48 h at 37 °C under standard assay conditions. Specific activity was recorded as glucose equivalents (µg/mL/h) per mg protein.

### pH and temperature optima and stabilities of enzyme mixtures

The pH optima were determined using 5 % (wet w/v) (0.5 % dry weight/v) apple pomace in 0.05 M universal buffer (boric, acetic and phosphoric acid) (Britton and Robinson 1931) at pH values ranging from pH 3.0–10.0. The temperature optima were determined using 5 % (wet w/v) apple pomace in citrate buffer (pH 5.0, 0.05 M) at temperatures ranging from 20–70 °C. The assays were performed in triplicate under standard assay conditions at 37 °C for 1 h. The final enzyme protein concentrations in the reaction mixture were 0.076 and 0.19 mg/mL for Viscozyme and Celluclast, respectively.

The pH stability of the Viscozyme and Celluclast solutions was determined by pre-incubating the enzyme solution in universal buffer (0.05 M) at pH 3.0, 4.0, 5.0 and 6.0 over a period of 24 h. Aliquots were removed at various time intervals and stored on ice. The standard assay was then performed in triplicate for the different time intervals for each pH. Suitable assay controls at each pH, where enzymes had not been pre-incubated with substrate, were included. The temperature stabilities of the Viscozyme and Celluclast samples were determined by incubating the enzyme solutions at 20, 28, 37 and 50 °C, respectively, over a period of 24 h. Aliquots were removed at various time intervals and stored on ice. The standard assay was then performed in triplicate for the different time intervals for each temperature. pH and temperature stability were also determined for a period of up to 15 days at pH 5.0 and 37 °C. Enzymes were pre-incubated in buffer (pH 5.0) at 37 °C, with aliquots taken at 1, 3, 6, 10 and 15 days, respectively. Enzyme hydrolysis was then performed at 37 °C for 1 h under standard assay conditions. The residual activities for each enzyme, and each pH and temperature, were calculated as a percentage of that of the controls (i.e. no pre-incubation).

### Determining the effect of temperature and buffering on apple pomace hydrolysis using a 50:50 combination of Viscozyme L and Celluclast 1.5L

The effect of temperature and pH on the activity of Viscozyme L - Celluclast 1.5L (50:50) (0.5 µL/mL, 0.038 mg/mL) combination was determined using 5 % apple pomace (wet w/v) (0.5 % dry w/v) final concentration and tap water. Three different incubation temperatures were investigated; namely room temperature (which fluctuated between 22 and 26 °C), 28 and 37 °C. The effect of buffering was investigated using tap water, deionised water and citrate buffer (0.05 M, pH 5.0) at 28 °C. The assays were performed in 1 L shake flasks (Labcon, Maraisburg, South Africa) and reactions were carried out for 72 h on a platform shaker at 165 rpm with 15 mL aliquots removed at different times for sugar analysis and pH determination.

### Effect of β-glucosidase supplementation

An initial substrate loading of 5 % apple pomace (wet w/v) (0.5 % dry w/v) was used, with fresh substrate (10 % apple pomace, 1 % dry w/v) added at 50 h intervals. One reactor contained only Viscozyme and Celluclast, while the other reactor also included Novozyme 188 (0.025 µL/mL, 0.0012 mg/mL) (10 % protein of Viscozyme and Celluclast).

### A comparison between fed-batch and batch reactors at high substrate loadings

A comparison between a fed-batch and a batch process was performed. Apple pomace was added in different ways to reactors, but each reactor had a final substrate concentration of 20 % apple pomace (wet, w/v) (2 % dry w/v). Three reactors were run, one starting with 5 % substrate, then 5 % additions at 6, 24 and 50 h, respectively. The other reactor was started with 10 % substrate, then 5 % additions at 24 and 50 h, respectively, and the last reactor was started with 20 % initial substrate loading with no further substrate additions. Viscozyme and Celluclast (50:50) (0.5 µL/mL, 0.038 mg/mL) were added together with Novozyme 188 (0.05 µL/mL, 0.0024 mg/mL) to all the reactors.

### The inhibitory effects of alcohols, sugars and lignin on Viscozyme and Celluclast

The effects of glucose, cellobiose, xylose and xylobiose at concentrations of 0–2 mM, organic acids at 1, 5 and 10 g/L, and metal ions at 10 and 50 mM in citrate buffer (pH 5, 0.05 M) on Viscozyme and Celluclast were determined. Assays were performed under standard conditions with a Viscozyme–Celluclast enzyme combination (50:50) (0.019 mg/mL for each enzyme) and also with individual enzymes for 24 h at 37 °C. An assay control with the enzymes and substrate, without the inhibitor, was also included. The residual activity for each enzyme or combination, at each inhibitor concentration, was then calculated as a percentage of that of the control (i.e. no inhibitor added).

## Results and discussion

Celluclast, Viscozyme and Biocip enzyme cocktails were tested in different combinations for activity on apple pomace. Selection was based on reports in the literature that indicated that Celluclast exhibited mainly cellulase activity; Viscozyme hemicellulase, arabinase, β-glucanase, cellulase and xylanase activities and Biocip polygalacturonase and cellulase activities. Combinations of Viscozyme and Biocip, or Celluclast and Biocip displayed a much lower yield of reducing sugars (glucose equivalents) than combinations of Viscozyme and Celluclast (See “Appendix”, Fig. 6). A combination of Viscozyme, Celluclast and Biocip displayed the same yields as the Viscozyme and Celluclast alone. These results indicated that a combination of Viscozyme and Celluclast was the best for the hydrolysis of apple pomace. Therefore, further experiments were performed using Celluclast and Viscozyme. Enzyme assays using different substrates were carried out to determine the enzyme activity profile for Celluclast and Viscozyme which are shown in Table 1.

**Table 1:** Activities of Viscozyme and Celluclast on different substrates

Substrate	Activity measured	Viscozyme	Celluclast
Carboxymethylcellulose	Endoglucanase	263.6 (±1.6)	385.1 (±2.4)
Birchwood xylan	Endoxylanase	191.1 (±1.5)	813.9 (±12.5)
Pectin	Pectinase	1177.3 (±28.3)	180.6 (±7.9)
Locust bean gum	Endomannanase	406.5 (±10.7)	124.4 (±0.8)
Polygalacturonic acid	Polygalacturonase	1470.7 (15.6)	149.6 (±2.7)
Filter paper	Total cellulase	33 (±0.3)	95.2 (±0.9)
4-Nitrophenyl-β-D-cellobioside	Cellobiohydrolase	0.004 (±0.0002)	0.03 (±0.001)
4-Nitrophenyl-β-D-glucopyranoside	β-D-glucosidase	0.2 (±0.001)	0.3 (±0.002)
4-Nitrophenyl-β-D-mannopyranoside	β-D-mannosidase	0.006 (±0.0002)	0.0003 (±0.0001)
4-Nitrophenyl-β-D-xylopyranoside	β-D-xylosidase	0.005 (±0.0002)	0.4 (±0.002)
4-Nitrophenyl-α-L-arabinofuranoside	α-L-Arabinofuranosidase	0.4 (±0.002)	0.06 (±0.001)

Activities are expressed as reducing sugar equivalents released (µg/ml/min) per mg protein (endoglucanase, endoxylanase, endomannanase, pectinase, polygalacturonase and total cellulose) and 4-nitrophenol liberated (µmol/ml/min) per mg protein (cellobiohydrolase, β-D-glucosidase, β-D-xylosidase, β-D-mannosidase, α-L-arabinofuranosidase)

Values are presented as mean values ± SD (*n* = 3)

Both mixtures contained similar activities, but in Viscozyme activity on pectin and polygalacturonic acid predominated, while Celluclast exhibited higher cellulase and endoxylanase activity. There are only a few reports in literature which indicate the different enzymes which are present in Celluclast, with no studies reporting on the enzyme activities in Viscozyme. Suwannarangsee et al. (2012) identified the proteins in Celluclast using LC/MS/MS and identified two cellobiohydrolases, five endoglucanases, one xyloglucanase, two β-xylosidases and two endoxylanases. However, they did not identify any pectinases and mannanases, which were detected through activity assays in our study, although Kovacs et al. (2009) reported the presence of mannanase and mannosidase activities in Celluclast. Suwannarangsee et al. (2012) were also able to identify the presence of the non-hydrolytic proteins Cip1, Cip2 and swollenin which contribute to hydrolysis but cannot be detected using the sugar assays.

The optimal enzyme ratio for hydrolysis was determined by combining different ratios of Viscozyme and Celluclast, while keeping the total protein concentration in the assay constant. The enzyme ratio that resulted in the highest release of reducing sugars was V50:C50 (925.5 U/mg protein), followed closely by V62.5:C37.5 and V75:C25 with 889.2 and 865.7 U/mg protein, respectively (See “Appendix”, Fig. 7). The enzyme combination of V50:C50 was selected for all subsequent experiments, although the results indicated that sugar release was not very sensitive to the specific ratio of the enzyme mixtures employed.

### Apple pomace composition

The composition of apple pomace was determined with respect to the main sugars and lignin. The amount of acid soluble lignin in apple pomace was 19.8 %, which fell in the range reported by other researchers, 15.2–20.4 % (Bhushan et al. 2008; Nawirska and Kwasniewska 2005). With respect to the neutral sugars in apple pomace, glucose concentration was the highest at 22.3 %, followed by arabinose (12.5 %) and galactose (5.1 %). Very little xylose was detected (1.1 %), but our analysis did not identify whether this formed part of xylan or another polysaccharide such as xyloglucan. The amount of glucose, arabinose and galactose obtained were similar to results reported by other researchers (Bhushan et al. 2008; Joshi and Attri 2006; Parmar and Rupasinghe 2013). The remainder (38 %) consisted mainly of galacturonic acid and other components in low concentrations, most likely ferulic acid, extractives, proteins, minerals and other sugars such as rhamnose which was not directly measured in our study. The HLPC method that was used in this study was unable to measure the galacturonic acid content directly, so that the actual percentage galacturonic acid could not be determined. The reported galacturonic acid content of apple pomace in the literature varies widely and we were unable to clarify this further, 49–64 % (Bhushan et al. 2008) and 11.7 % (Nawirska and Kwasniewska 2005). None of the available methods for determination of lignocellulose composition in the literature have been developed for high pectin-containing substrates, making an accurate mass balance and calculation of yields for galacturonic acid difficult. The presence of starch in the apple pomace was also determined. There was no starch detected in the apple pomace.

### Effect of temperature and pH (buffering) on apple pomace hydrolysis

Two of the most important reaction conditions for enzyme hydrolysis are temperature and pH. We first determined the temperature and pH optima for Celluclast and Viscozyme on apple pomace as a substrate and found that Celluclast and Viscozyme both displayed a broad pH range (data not shown). Viscozyme displayed optimal activity over a wide pH range of 3.0–6.0, while Celluclast displayed optimal activity over a narrow range of pH of 3.0–4.5. Viscozyme was very stable and had a residual activity of more than 80 % at pH 4.0, pH 5.0 and pH 6.0 after 24 h incubation, which decreased to around 60 % at pH 3.0. Celluclast had a residual activity of more than 80 % at pH 3.0–6.0 after 24 h incubation, with the highest stability observed at pH 4.0. This indicated that the two mixtures will function effectively over a pH range between pH 3.0 and pH 6.0.

The temperature optima for both commercial enzyme preparations were 50 °C (See “Appendix”, Fig. 8). However, Celluclast had a broad temperature range from 25 to 60 °C, while Viscozyme displayed a range of activity from 25 to 55 °C with two peaks at 37 and 50 °C, respectively. High levels of activity were present at the temperatures used in this study. With respect to stability, Viscozyme retained residual activity above 90 % after 24 h incubation at 20, 28 and 37 °C. However, the residual activity decreased to about 60 % at 50 °C. Celluclast maintained residual activity of above 90 % at 20, 28, 37 and 50 °C, with the greatest stability observed at 37 °C. A combination of Viscozyme and Celluclast was more stable than the individual enzyme mixtures and displayed more than 90 % residual activity after 15 days at 37 °C and pH 5.0 (data not shown).

**Fig. 1:**
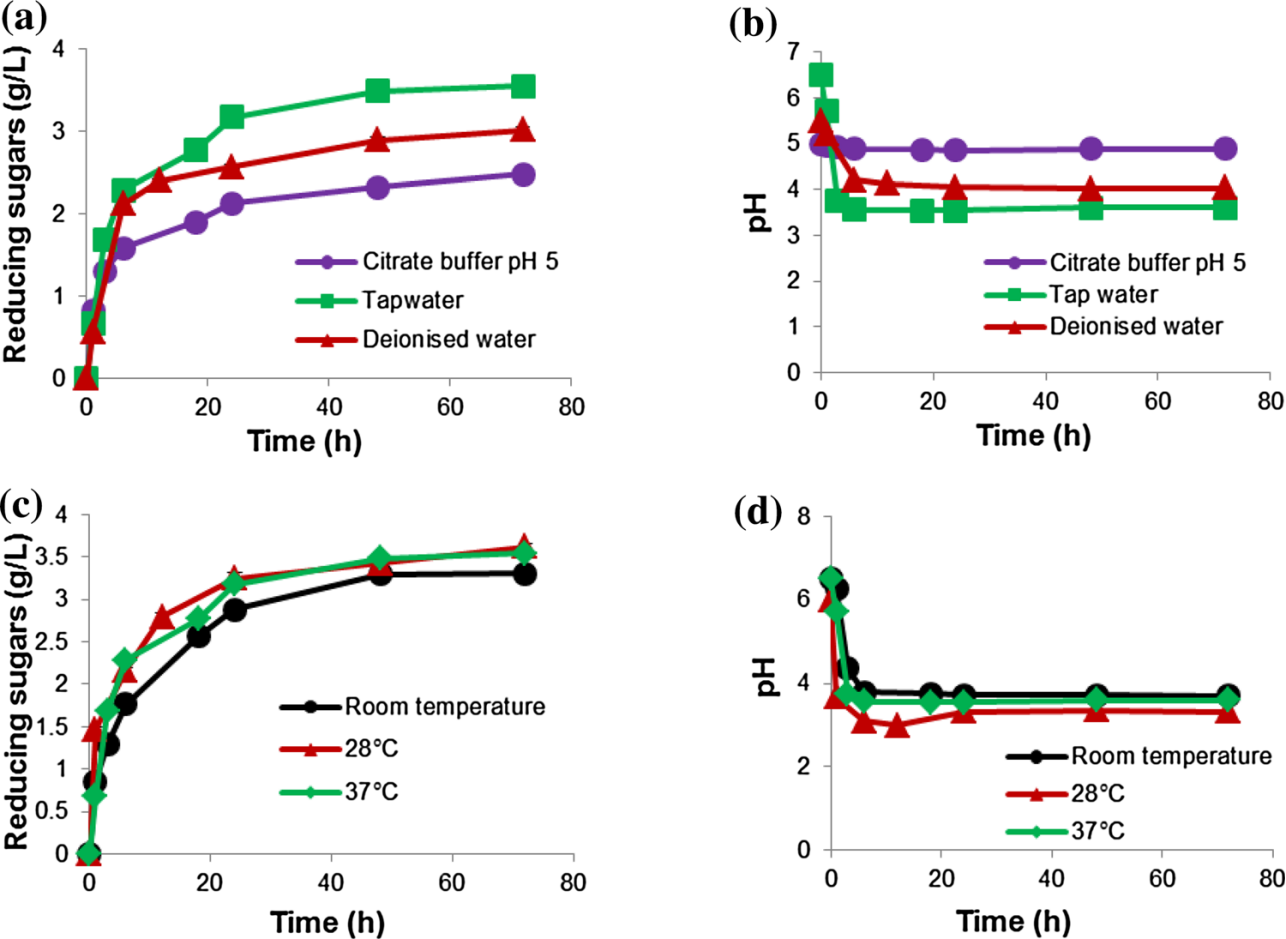
Evaluation of the effect of temperature and pH. The amount of reducing sugars released and pH measured at different time intervals using citrate buffer pH 5.0, tap water and deionised water at 28 °C (**a**, **b**); and different incubation temperatures using tap water (**c** and **d**), respectively. Apple pomace 5 % (wet, w/v) (0.5 % dry w/v) was used as substrate in shake flasks of 1 L reaction volumes and was mixed by shaking on a platform shaker for 72 h. Data points are presented as mean values ± SD (*n* = 3)

To determine the effect of buffering on apple pomace hydrolysis, the amount of reducing sugars released and the change in pH were measured over a period of 72 h using citrate buffer, tap water and deionised water and different temperatures. The results are shown in Fig. 1. There was a rapid release of reducing sugars during the first 6 h with the unbuffered reactions showing the highest levels, coinciding with a sharp decrease in pH observed during this time (Fig. 1a, b).

Tap water showed the highest release of reducing sugars (3.54 g/L) followed by deionised water (3.01 g/L) and lastly, citrate buffer (pH 5.0) (2.47 g/L). Citrate buffer maintained the pH around pH 5.0, while in the case of tap water and deionised water, the pH dropped to around pH 3.6 (Fig. 1b). The drop in pH in the unbuffered reactions can be attributed to the release of acids such as galacturonic acid from the pectin component. The higher levels of reducing sugars released in reactions (where the pH displayed a significant decrease) indicated that the multiple enzymes present in the mixture probably displayed activity at different pH values. The change in pH therefore seems to promote the activity of certain enzymes. This seems to be supported by the wide range in pH optima for the mixtures. Achieving the best result without buffering means that the costs associated with adding chemicals are significantly reduced.

The difference in results between tap water and deionised water is difficult to explain. One hypothesis is that the presence of metal ions in tap water that may have a stimulatory effect on the enzymes, e.g. Ca^2+^, Mn^2+^, Mg^2+^ and K (Ferchak and Pye 1983; Tejirian and Xu 2010). However, when the effect of metal ions was examined on enzyme activity, no enhancement in activity was found (data not shown).

Although the optima for the enzyme mixtures were 50 °C, the aim was to limit the energy and chemical inputs and associated costs and therefore decided to test the rate of hydrolysis at room temperature (no energy input, with fluctuations), 28 °C (minimal energy input but constant temperature) and 37 °C. Our results also indicated that the enzymes displayed greater stability at lower temperatures. Reactions at all three temperature conditions did not display large differences in terms of reducing sugar release and pH (Fig. 1c and d) using an unbuffered system (i.e. tap water). The amount of reducing sugars released after 72 h at room temperature, 28 and 37 °C were 3.3, 3.6 and 3.5 g/L, respectively. Although the reaction at room temperature produced slightly lower amounts of reducing sugars, such conditions could still be effective for bioreactor applications, since no input of energy and cost associated with buffering was required, while still obtaining high levels of sugars. Depending on the value of the products produced in an industrial process, a temperature of 50 °C can still be implemented and reactions can be run for shorter times.

### Effect of β-glucosidase supplementation

**Fig. 2:**
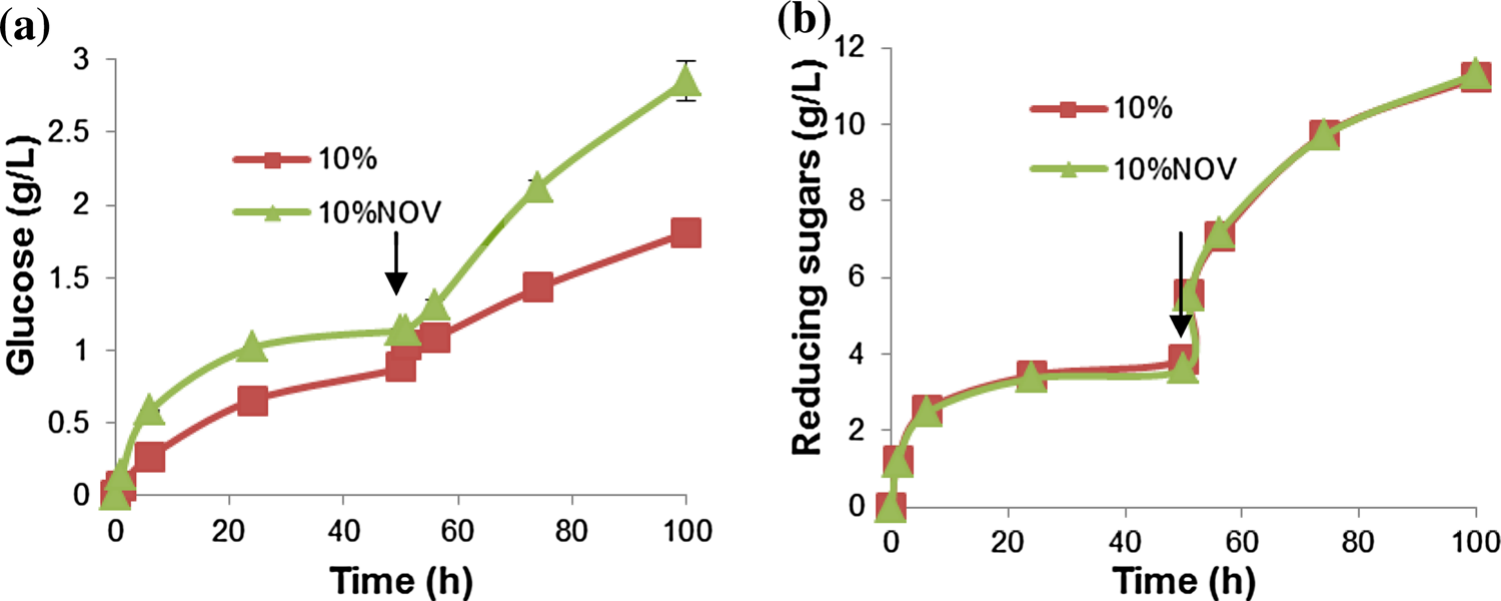
Evaluation of β-glucosidase supplementation. Concentration of sugars released at different time intervals for the fed-batch reactor mixed with compressed air at room temperature, with (10 %Nov) and without Novozyme 188 supplementation (10 % protein) (**a**—glucose,** b**—reducing sugars). Both reactors started with 5 % (wet, w/v) (0.5 % dry w/v) substrate and 10 % substrate (1 % dry w/v) added every 50 h (indicated by *arrow*). Data points are presented as mean values ± SD (*n* = 3)

The effect of supplementing with additional β-glucosidase activity was determined by adding Novozyme 188 to the enzyme mixture at 10 % (protein weight) and comparing it to a reaction without Novozyme 188 supplementation. The initial substrate concentration for both reactors was 5 % and additional 10 % substrate was added every 50 h. The amount of glucose and reducing sugars released are shown in Fig. 2.

The amount of glucose released from bioreactors with 10 and 10 %Nov substrate loadings after 100 h was 1.8 and 2.9 g/L, respectively, giving overall glucose yields of 69 % (at 50 h) and 45 % (at 100 h) using 10 % substrate loadings without Novozyme addition. The glucose yield was 88 % (50 h) and 70 % (100 h) using 10 % substrate loading and supplementation with Novozyme (10 %Nov). After 200 h, the amount of glucose released with 10 and 10 %Nov increased to 2.1 and 4.7 g/L, respectively (See “Appendix”, Fig. 9). The amount of reducing sugars released from 10 and 10 %Nov bioreactors was 11.2 and 11.3 g/L, respectively, which corresponded to yields of 74.5 % (at 50 h) and 69.4 % (at 100 h) using 10 % substrate loading without Novozyme addition. The reducing sugar yield was 69 % (50 h) and 68 % (100 h) when using 10 % substrate loading with Novozyme supplementation. The amount reducing sugars increased to 21.2 and 23.0 g/L for bioreactors with 10 and 10 %Nov, respectively (See “Appendix”, Fig. 9). β-glucosidase cleaves cellobiose into glucose and can therefore increase the yield of glucose, but, more importantly, prevents end-product inhibition of cellulases by cellobiose (Teeri 1997). Our studies showed significant inhibition of Viscozyme L and Celluclast 1.5L in the presence of cellobiose which indicates that end-product inhibition is an important factor (see Fig. 5), while the increased release of glucose in the presence of Novozyme 188 also confirms that β-glucosidase activity resulted in cleavage of cellobiose. Only a small difference in reducing sugars produced was observed in the two treatments (Fig. 2b) indicating that Novozyme 188 supplementation had no effect on the release of reducing sugars. However, the effect on glucose release became significant after 50 h hydrolysis. This indicated that Novozyme 188 supplementation became significant over longer hydrolysis times and when substrate loading was high.

### A comparison between fed-batch and batch reactors at high substrate loadings

**Fig. 3:**
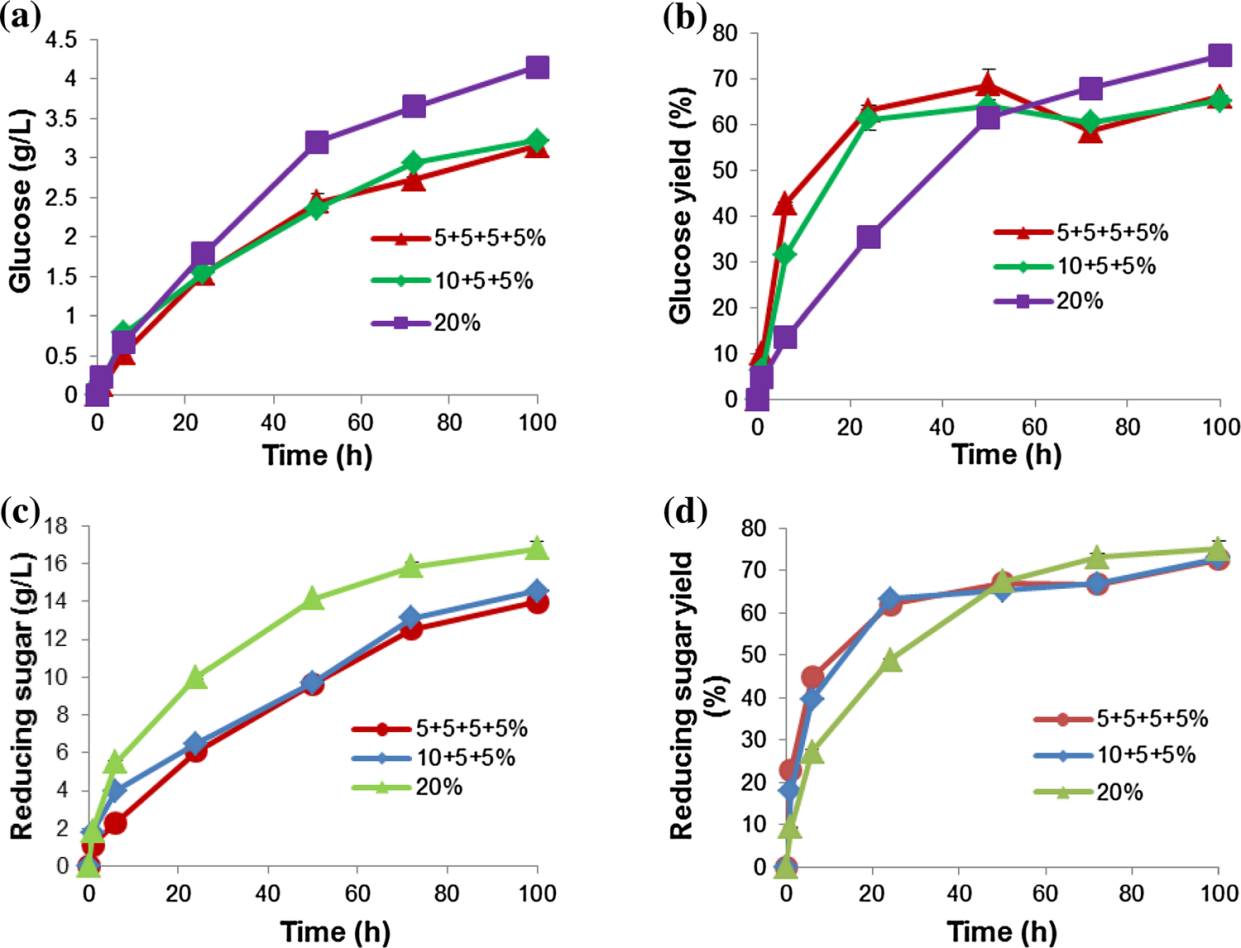
Evaluation of different substrate loadings. Concentration of sugars released at different time intervals for the reactor mixed with compressed air at room temperature with different substrate loading regimes. Reactors are identified according to their initial substrate loading (5, 10 and 20 % wet w/v) (0.5, 1 and 2 % dry w/v). **a** (Glucose), **b** (glucose yield), **c** (reducing sugars) and **d** (reducing sugar yield). Data points are presented as mean values ± SD (*n* = 3)

Further studies were carried out to compare a fed-batch system with a batch process at a high substrate loading (20 % wet w/v). Three reactors with different substrate feeding regimes were compared and the results are shown in Fig. 3. The final substrate loading for each reactor was 20 %.

The amounts of glucose (% yield) released by the different reactor treatments (5, 10 and 20 % starting substrate concentration) after 100 h were 3.2 (66.1 %), 3.2 (65.5 %) and 4.2 g/L (75 %), respectively (Fig. 3a and b). The bioreactors (5, 10 and 20 % substrate) produced 14 g/L (72.7 %), 14.6 g/L (73.1 %) and 16.8 g/L (75 %) reducing sugars, respectively (Fig. 3c, d). Reactors that started with 5 and 10 % substrate performed almost identically. However, the reactor operated in batch mode, starting with 20 % substrate, produced a higher sugar concentration and yield after 100 h (Fig. 3).

**Fig. 4:**
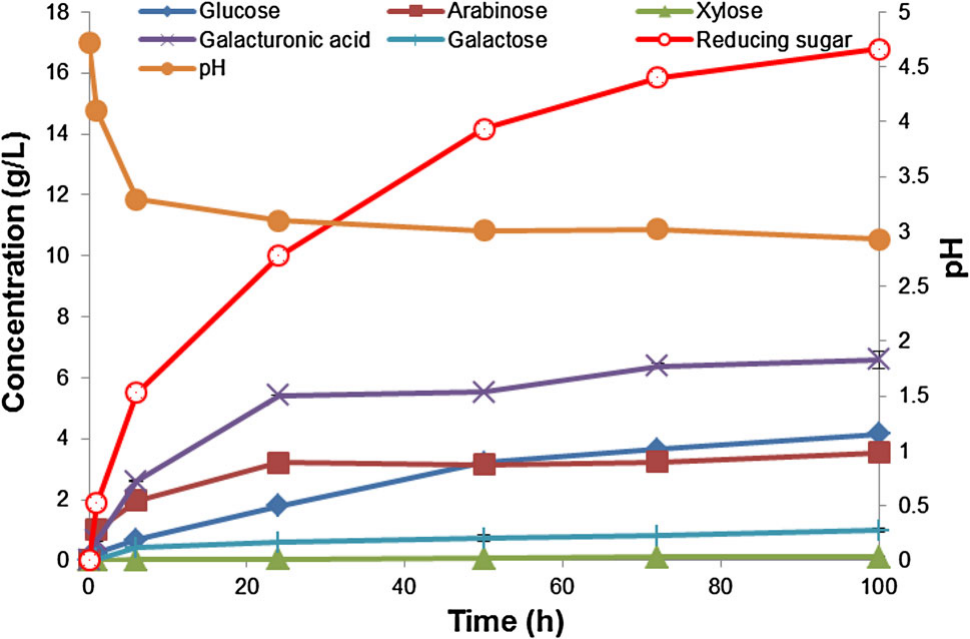
Concentration of sugars released at different time intervals for the batch reactor mixed with compressed air at room temperature. The initial substrate loading 20 % (wet, w/v) (2 % dry w/v). Data points are presented as mean values ± SD (*n* = 3)

A detailed sugar profile for the batch reaction at 20 % substrate loading is shown in Fig. 4.

Galacturonic acid (6.6 g/L, 78 % yield), followed by glucose (4.2 g/L, 75 % yield), arabinose (3.5 g/L, 90 % yield) and galactose (1.0 g/L, 87 % yield) were the major products released from apple pomace (Fig. 4). Galacturonic acid, from the pectin backbone, and arabinose and galactose, from neutral side chains in pectin, were rapidly released in the first 6 h, as compared to glucose and xylose which showed a slower release and indicated that the enzymes seemed to hydrolyse the pectin component first (Fig. 4). In an unlikely situation for apple pomace, galacturonic acid could also be released from xylan side chain substitution, and arabinose and galactose could also be from xyloglucan and xylan. We did not specifically assay for arabinase and galactanase activity, but it appears that these enzymes were present in the commercial mixtures as Voragen et al. (2009) clearly showed that apples contained high levels of neutral side chains. It is therefore unlikely that the high concentrations of arabinose and galactose were released by the actions of other enzymes, such as α-arabinofuranosidase or α-galactosidase, although such enzymes may have a contribution.

Pectinases, arabinases and galactanases appeared to be more active at the initial stages, modifying and opening up the structure for cellulases to hydrolyse the cellulose component and release glucose. Some studies have indicated that pectin and the neutral side chains in pectin may mask the access of cellulases to their substrate in a similar manner to xylan and that the synergistic action of these enzymes may contribute to the release of glucose (Pakarinen et al. 2012; Spagnuolo et al. 1997; Van Dyk et al. 2013). This will be investigated in future studies.

There seemed to be a direct correlation between the release of galacturonic acid and the decrease in pH, confirming results from Fig. 1. Figure 4 indicated that most of the sugars were released within the first 50 h; therefore, operating the reactors for a shorter time in a batch mode appears to be effective.

The products released from apple pomace can be used for producing value-added commodities or products. The production of multiple sugars and galacturonic acid poses a challenge for bioethanol production as there is not a single organism capable of fermenting all these sugars. Glucose can be fermented to ethanol by *Saccharomyces cerevisiae* and *Zymomonas mobilis*, while arabinose, xylose and galacturonic acid can be fermented using genetically modified organisms, e.g. *E. coli* K011 and *Erwinia* species (*E. crysanthemi* EC16 and *E. carotovora* SR38) (Dien et al. 2003; Grohmann et al. 1995, 1998; Kang et al. 2014). Galacturonic acid can also be utilised in the synthesis of tensioactive agents for pharmaceutical and cosmetic purposes, as an acid agent, and for the production of vitamin C (Baciu and Jordening 2004; Boluda-Aguilar et al. 2010; Pourbafrani et al. 2010). Arabinose can be used for diagnostics (in bacteriology); derivatised to 5-deoxy-L-arabinose, which has anti-Parkinson properties; and as a precursor of l-fructose and l-glucose that are used as sweeteners (Baciu and Jordening 2004). Xylose and glucose can also be utilised in the food industry in the production of food sweeteners, xylitol and sorbitol, respectively (Bhushan et al. 2008; Demirbas 2008). However, there may be other sugars and products in the hydrolysate that were not measured and these may also add to the value of degraded apple pomace.

### Influence of sugars and organic acids on Viscozyme L and Celluclast 1.5L

**Fig. 5:**
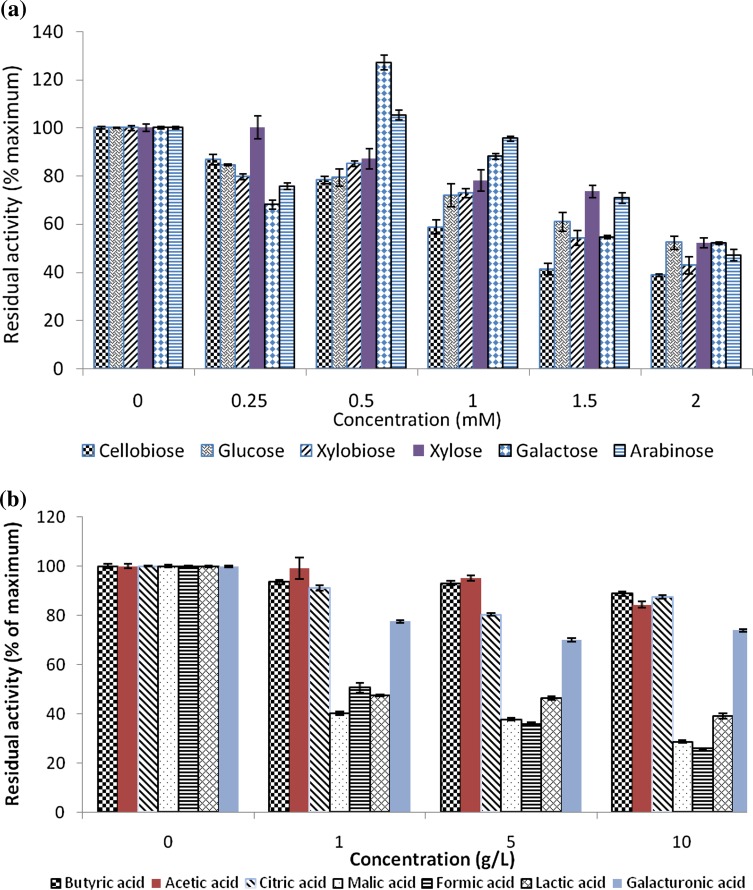
Viscozyme and Celluclast (50:50 ratio) residual activity in the presence of different sugars (**a**); and different organic acids (**b**). Residual activity was calculated as the activity obtained at each acid concentration divided by activity with no acid included, which was then multiplied by 100. Activity was measured using 5 % (wet w/v) (0.5 % dry w/v) apple pomace as substrate. Data points are presented as mean values ± SD (*n* = 3)

**Fig. 6:**
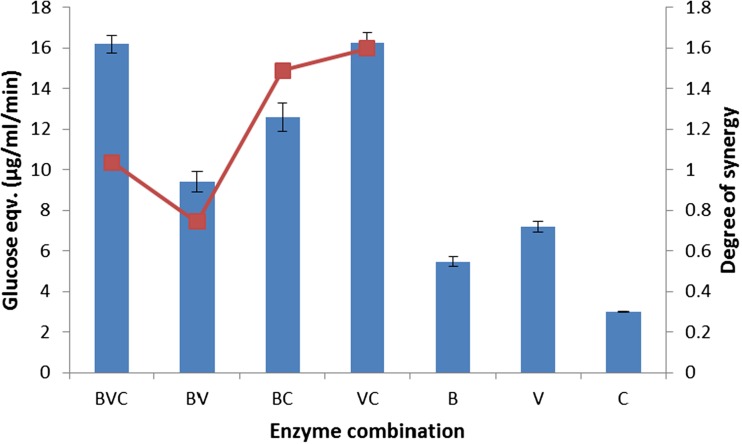
Activities (*solid bars*) and degree of synergy (*line*) of Biocip membrane (*B*), Viscozyme L (*V*) and Celluclast 1.5L (*C*) individually and their combinations (equal ratios) on 5 % (wet w/v) apple pomace after 1 h incubation at 37 °C. Degree of synergy was calculated as the activity of the enzyme combination divided by the sum of the individual enzyme activities. Data points are presented as mean values ± SD (*n* = 3)

**Fig. 7:**
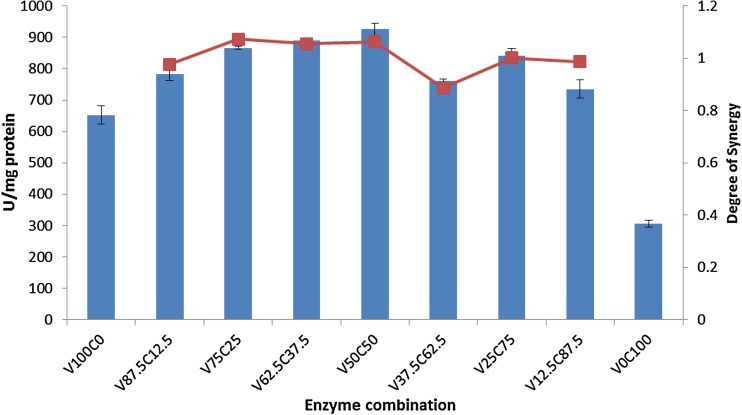
Activity (*solid bars*) and degree of synergy (*line*) of different ratios of Viscozyme L (V) and Celluclast 1.5L (C) on 5 % (wet w/v) apple pomace at 37 °C, pH 5.0 for 48 h. Data points are presented as mean values ± SD (*n* = 3)

**Fig. 8:**
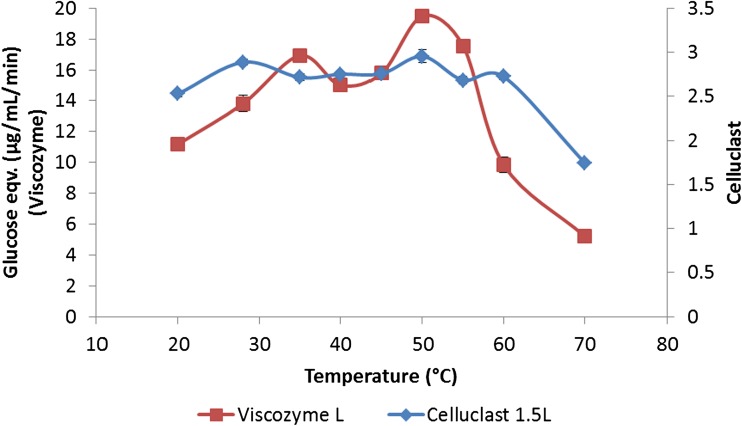
Temperature optima for Viscozyme L and Celluclast 1.5L activity. Activity was measured on 5 % (wet w/v) apple pomace using a temperature range of 20–70 °C and a citrate buffer at pH 5.0. Data points are presented as mean values ± SD (*n* = 3)

**Fig. 9:**
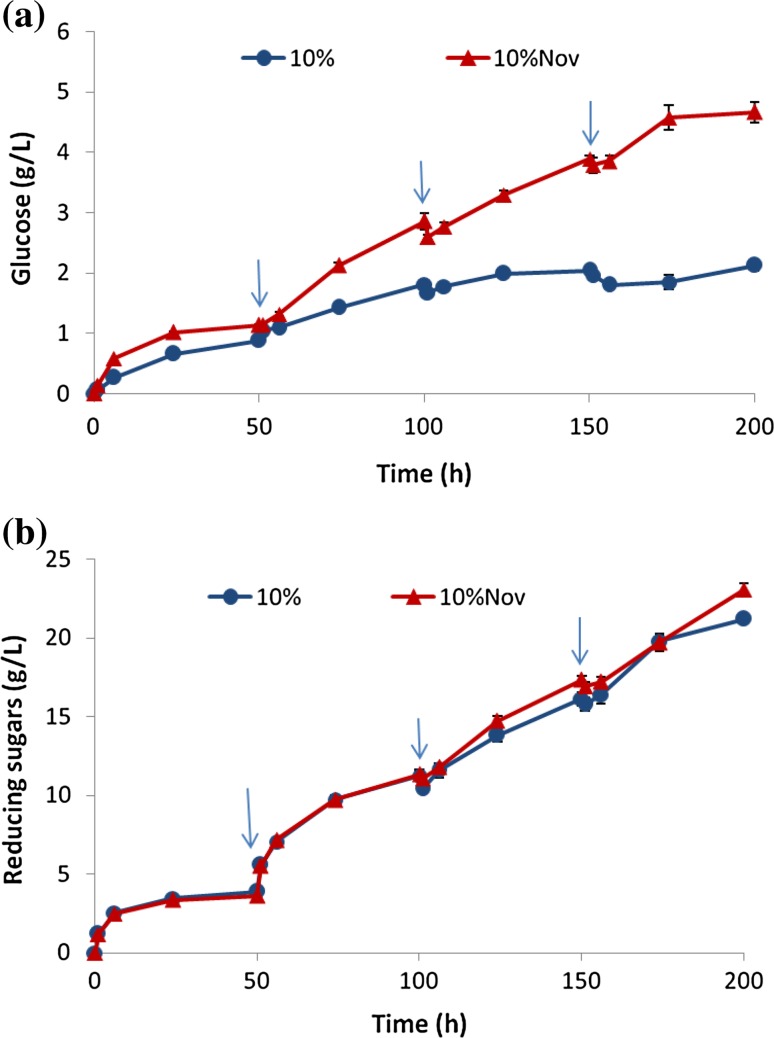
Concentration of sugars released at different time intervals for the fed-batch reactor mixed with compressed air at room temperature, with (10 %Nov) and without Novozyme 188 supplementation (10 %) (**a**—glucose, **b**—reducing sugars). Both reactors started with 5 % (wet, w/v) substrate and 10 % substrate added every 50 h. Data points are presented as mean values ± SD (*n* = 3)

The effect of sugars on the Viscozyme and Celluclast combination was measured to determine their possible impact on the hydrolysis performance and is displayed in Fig. 5.

Hydrolysis of cellulose and hemicellulose results in the production of sugars such as glucose, cellobiose, cello-oligomers, xylose, xylo-oligomers, galactose and arabinose; hence some of these sugars were chosen for this study (Fig. 4). For the Viscozyme:Celluclast (50:50) combination, all the sugars tested displayed a linear trend of inhibition with increased concentrations of the inhibitor, although arabinose and galactose displayed activation at very low concentrations. Cellobiose and xylobiose displayed greater inhibition compared to the monomers glucose, xylose, arabinose and galactose. At high concentrations (2 mM), activity was reduced to ≈40 % in the presence of cellobiose and xylobiose, while activity was about ≈50 % in the presence of glucose and xylose. The sugar concentrations in the bioreactor after 100 h hydrolysis (Fig. 4) were above 2 mM (except for xylose) and therefore are inhibitory to the enzymes slowing down the hydrolysis process. However, these sugars are released gradually in the bioreactor as compared to the initially high amounts for inhibition studies plus additional sugars released during hydrolysis. Using β-glucosidase in 100 h bioreactor experiments, the inhibitory effect of glucose was minimised. In order to avoid inhibitory effect of the sugars on the enzymes during hydrolysis, simultaneous saccharification and fermentation (SSF) can be implemented (Kang et al. 2014). Membrane bioreactors can also be used (Andric et al. 2010; Baeyens et al. 2015).

Organic acids may be present in a bioreactor for different reasons. They may be produced through a fermentative pathway or through hydrolysis of the substrate—for example galacturonic acid is released from the degradation of apple pomace degradation (Gullon et al. 2008). If SSF and consolidated bioprocessing (CBP) bioreactors is used, then the organic acids released from fermentation can inhibit saccharifying enzymes. The organic acids may also inhibit microbial fermentation by inhibiting their growth and metabolism (e.g. formic acid and acetic acid have been reported to have an inhibitory effect on microbial fermentation) (Parmar and Rupasinghe 2012). Butyric, acetic and citric acid only had a slightly inhibitory effect on enzyme activity, with activity remaining above 80 %, even at concentrations of 10 g/L. Activity of 70–80 % was retained at the concentrations of galacturonic acid tested (1–10 g/L). The results observed in Fig. 4, which showed a concentration of 6.6 g/L galacturonic acid released after 100 h hydrolysis, indicated there may have been some enzyme inhibition taking place. However, in enzyme hydrolysis the concentration of galacturonic acid increases gradually as compared to initially high concentrations for inhibition studies. The concentration of galacturonic acid in the inhibition studies increases above the initial concentrations due to extra galacturonic acid released by enzyme action. Malic, formic and lactic acid displayed the greatest inhibitory effect and the enzyme activity decreased to below 50 %, even at low concentrations of the organic acids (1 g/L). However, these acids will affect the hydrolytic enzymes in the bioreactor if SSF is used. Considering these results, separate hydrolysis and fermentation will be more ideal to prevent enzyme inhibition in industrial bioreactors.

## Conclusions

The obtained results indicated that hydrolysis of apple pomace using Viscozyme L and Celluclast 1.5L can be successfully performed at room temperature without buffering, therefore lowering the operational costs involved in the treatment of this waste, while producing multiple products that can further contribute in value addition. β-glucosidase supplementation to Viscozyme L and Celluclast 1.5L was very important as it resulted in the doubling of the amount of glucose released over longer periods of incubation. The best results were obtained by operating the bioreactors in a batch mode producing 4.2 g/L glucose and 16.8 g/L reducing sugar (75 % yield for both glucose and reducing sugar) compared to the fed-batch bioreactors. Apple pomace hydrolysis products such as glucose, galacturonic acid, arabinose and galactose (75, 78, 90, and 87 % yield, respectively) can be further explored for value addition, making the treatment process more cost-effective. This study revealed that there were multiple and complementary enzymes present in both the Viscozyme and Celluclast cocktails. Employing similar hydrolysis conditions for the enzymes allow for the cost-effective application in industrial bioreactors. The inhibitory effect of various sugars on Viscozyme and Celluclast can be minimised using a SSF or a membrane bioreactor system.

## Appendix

See Figs. 6, 7, 8 and 9.
